# 

**Published:** 1906-04-28

**Authors:** 


### Medical Appointments and Vacancies.

### VACANCIES.

Barbados General Hospital.—Junior Resident Surgeon.

Buxton, Derbyshire, Devonshire Hospital.—Assistant House Surgeon.

Carlisle Non-Provident Dispensary.—Resident Medical Officer.

Charing Cross Hospital.—Assistant Physician.

Cronstadt, Seamen's Hospital.—Resident Medical Officer.

Douglas, Isle of Man, Noble's Isle of Man Hospital and Dispensary.—Resident House Surgeon.

East Suffolk and Ipswich Hospital.—Third House Surgeon.

Lancaster, Royal Albert Asylum.—Junior Assistant Medical Officer.

Leicester Infirmary.—Assistant Surgeon.

Montrose Royal Asylum, Sunnyside, Montrose.—Senior Assistant Medical Officer.

Newport and Monmouthshire Hospital.—Junior Resident Medical Officer.

Oldham Infirmary.—Junior House Surgeon.

Oxford, Littlemore Pauper Lunatic Asylum.—Second Assistant Medical Officer.

Swansea General and Eye Hospital.—House Surgeon.

Warrington Infirmary and Dispensary.—Junior House Surgeon.

West Bromwich District Hospital.—Resident Assistant House Surgeon.

West London Hospital, Hammersmith Road, W.—Assistant Anæsthetist. Also Second Assistant Anæsthetist. Also Casualty Surgeon.

**NOTICE TO CORRESPONDENTS.**

All MSS., Letters, Books for Review, and other matters intended for the Editor should be addressed The Editor, "The Hospital," The Hospital Buildings, 28 & 29 Southampton Street, Strand, W.C.

The Editor cannot undertake to return rejected MS., even when accompanied by stamped directed envelope.

**Notice to Advertisers and Subscribers.**

All Advertisements, Orders for Copies of The Hospital, and Business Communications should be addressed to **THE MANAGER (Not to the Editor),** The Hospital, 28 & 29 Southampton Street, Strand London, W.C.

The Cost of Subscription, post free, is as follows:—Per week, 3½d.; per quarter, 3s. 3d.; per half-year, 6s. 6d.; per annum, 13s. Foreign and Colonial:—Per annum, 19s. 6d., payable in all cases, in advance.

### HALL MARK

upon an article of gold or silver is an absolute guarantee of its quality. Equally so is

**this** 

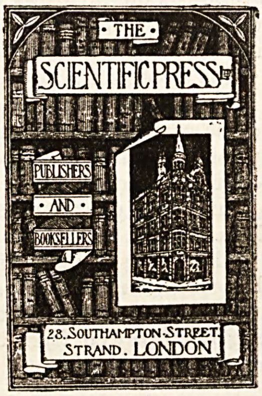

**Mark**

on a Nursing Book a proof that it is written by a competent authority on the subject of which it treats—that it is, in fact, the best book of its kind, whether as a Text-book or as a work of reference.

### These are some of our Books

**NURSING: ITS PRINCIPLES AND PRACTICE.**
By ISABEL ADAMS HAMPTON.
New and Enlarged Edition. Illustrated.
**7s. 6d.** net; **7s. 10d.** ost free.

**ELEMENTARY ANATOMY AND SURGERY FOR NURSES.**
By WILLIAM McADAM ECCLES, M.S. Lond., M.B. F.R.C.S., L.R.C.P. Profusely Illustrated. **2s. 6d.** post free.

**ELEMENTARY PHYSIOLOGY FOR NURSES.**
By C. F. MARSHALL, M.D., B.Sc., F.R.C.S.
Illustrated. **2s.** ost free.

**ELEMENTS OF ANATOMY AND PHYSIOLOGY.**
By W. BERNARD SECRETAN, M.D..
Profusely Illustrated. **2s.** net; **2s. 3d.** t fre .

**SURGICAL WARD-WORK AND NURSING.**
By ALEXANDER MILES, M.D. Edin., C.M., F.R.C.S .
Second Edition. Enlarged and entirely Revised. Copiousl Illustrated. **3s. 6d.** net; **3s. 10d.** post free.

**HOSPITAL SISTERS AND THEIR DUTIES.**
By EVA C. E. LÜCKES, Matron, London Hospital.
Third Edition. Enlarged and partly re-written.
**2s. 6d.** post free.

**THE MODERN NURSING OF CONSUMPTION.**

By Dr. JANE H. WALKER. **1s.** net; **1s. 2d.** post free.

**OPHTHALMIC NURSING.**
By SYDNEY STEPHENSON, M.B., F.R.C.S.E.
New and Revised Edition. Profusely Illustrated with Original Drawings, List of Instruments required, and a Glossary of Terms.
**3s. 6d.** net, **3s. 10d.** o f e

**NURSING IN DISEASES OF THE THROAT, NOSE, AND EAR.**
By P. MACLEOD YEARSLEY, F.R.C.S. Eng., M.R.C.S., L.R.C.P. Lond. Illustrated. **2s. 6d.** post free.

**ART OF MASSAGE.**
By A. CREIGHTON HALE.
Second Edition, profusely Illustrated with over 70 special Cuts.
**6s.** post fr e.

**ART OF FEEDING THE INVALID.**
By a MEDICAL PRACTITIONER and a LADY PROFESSOR OF COOKERY.
New and Popular Edition, with Special Chapter on Diet or Consumptives. **1s. 6d.** post free.

**DIET IN SICKNESS AND IN HEALTH.**
By Mrs. ERNEST HART. With an Introduction by Sir Henry Thompson, F.R.C.S., M.B. Lond.
Illustrated. **3s. 6d.** post free.

*We shall be pleased to send you free of cost on receipt of post-card a copy of our Catalogue, which contains many other works on various Nursing subjects equally as good as the above books.*

**The Scientific Press, Ltd.,**

***Catalogue of Nursing Manuals sent post free on application.***

**Publishers of Nursing Text-Books, Charts, and Case-Books of all Descriptions.**

**28 & 29 SOUTHAMPTON STREET, STRAND, LONDON, W.C.**

### YOUR INFANT WILL THRIVE:

NEAVE'S FOOD is a PERFECTLY SAFE FOOD for the most delicate child if used according to instructions. It assists teething, is a valuable bone-former, relieves constipation, and is a complete diet for Infants.

### Neave's Food

Write for "Hints about Baby," by a Trained Nurse — postcard to JOSIAH R. NEAVE & CO., Fordingbridge, *via* Salisbury.

Purveyors by Special Appointment to **H.I.M. THE EMPRESS OF RUSSIA.**

"An invaluable handbook for all engaged in philanthropic work."
ST. JAMES'S GAZETTE.

### BURDETT'S HOSPITALS & CHARITIES, 1906.

BEING

The Year Book of Philanthropy and the Hospital Annual.

Over 1,000 pages.

Price **6/-** net; **6/5** post free.

*Of all Booksellers, or of*

THE SCIENTIFIC PRESS, Ltd.,
28 and 29 Southampton Street, Strand, London, W.C.

